# Age at Menarche and Risk of Colorectal Cancer: A Meta-Analysis

**DOI:** 10.1371/journal.pone.0065645

**Published:** 2013-06-06

**Authors:** Chun-Yan Li, Bo Song, Ying-Yan Wang, Hua Meng, Shi-Bin Guo, Li-Na Liu, Hai-Chen Lv, Qi-Jun Wu

**Affiliations:** 1 Department of Gastroenterology, the First Affiliated Hospital of Dalian Medical University, Dalian, China; 2 Department of Pathology, Dalian Medical University, Dalian, China; 3 Laboratory Center for Diagnostics, Dalian Medical University, Dalian, China; 4 Fudan University, Shanghai, China; 5 Department of Epidemiology, Shanghai Cancer Institute, Renji Hospital, Shanghai Jiaotong University School of Medicine, Shanghai, China; MOE Key Laboratory of Environment and Health, School of Public Health, Tongji Medical College, Huazhong University of Science and Technology, China

## Abstract

**Background:**

Various observational studies have focused on the relationship between menarcheal age and the risk of colorectal cancer (CRC). However, the association is still controversial because of inconsistent results. Therefore, we performed a meta-analysis to assess this issue from epidemiological studies.

**Methods:**

After a literature search in MEDLINE, EMBASE, and Web of Science for studies of menarcheal age and CRC risk published through the end of January 2013, we pooled the relative risks (RRs) from included studies using a fixed- or random-effects model and performed heterogeneity and publication bias analyses. All statistical tests were two-sided.

**Results:**

Eleven case-control and 11 cohort studies were eligible for inclusion in our analysis. The random-effects pooled RR for oldest versus youngest menarcheal age was 0.95 [95% confidence intervals (CIs) = 0.85–1.06], with significant heterogeneity (*Q* = 61.03, *P*<0.001, *I* ^2^ = 65.6%). When separately analyzed, case-control (RR = 0.95, 95% CI = 0.75–1.21) and cohort studies (RR = 0.97, 95% CI = 0.90–1.04) yielded similar results. Moreover, similar results were also observed among the subgroup analyses by study quality, population, exposure assessment, anatomic cancer site, subsite of colon cancer, and several potential important confounders and risk factors. There was no evidence of publication bias and significant heterogeneity between subgroups detected by meta-regression analyses.

**Conclusions:**

Findings from this meta-analysis demonstrated that menarcheal age was not associated with the risk of CRC in humans. Further studies are warranted to stratify results by the subsite of colon cancer and menopause status in the future.

## Introduction

Colorectal cancer (CRC) is the third most common type of cancer, with 1.23 million new cases diagnosed in 2008 worldwide, accounting for almost 9.7% of all cases of cancer [1]. Ecological studies, migrant studies, and secular trend studies have provided evidence that environmental risk factors are of major importance in the cause of CRC [2], [3]. Observational and experimental studies have also suggested that sex hormones, particularly estrogen, may play a protective role in colorectal carcinogenesis either indirectly by reducing secondary bile acids and insulin-like growth factor-I (IGF- I) or directly by regulating cell growth in the colonic epithelium and inhibiting cell proliferation of colorectal tumors by binding to the estrogen receptor [4]. Moreover, several systematic reviews and meta-analyses reported that some reproductive factors including oral contraceptives use and hormone replacement therapy might reduce the risk of CRC [5], [6].

Menarche is not only the milestone of puberty initiation but the initiation of hormone changes in the childhood and adolescent period. Furthermore, age at menarche also has been used as surrogate marker for lifetime exposure to endogenous estrogens. Several recent meta-analyses demonstrated that later menarcheal age was inversely associated with the risk of ovarian and breast cancer [7], [8]. However, the epidemiological evidence for a causal link between menarcheal age and CRC risk has been inconsistent. Some studies have suggested inverse associations [9], [10], [11], [12], whereas others have found positive or no association [13], [14], [15], [16], [17]. When results are stratified by site of CRC or subsite of colon cancer, a clear pattern in the association still has not been strongly evident [9], [12], [18], [19]. Therefore, to further clarify the association between menarcheal age and the risk of CRC, we performed a comprehensive review and meta-analysis including published observational studies up to January 2013.

## Methods

### Literature search strategy

We conducted a literature search including published studies from database initiation until January 31, 2012 using the MEDLINE (PubMed), EMBASE, and ISI Web of Science database. The search was limited to published studies in English and studies of humans using the following search key words and medical subject heading terms: (menarche OR reproductive OR reproduction OR reproductive factors) AND (colorectal OR colorectum OR colon OR rectal OR rectum) AND (cancer OR neoplasm OR carcinoma OR tumor). Furthermore, we also reviewed the references of all included studies for additional publications. We then adhered to standard criteria for conducting and reporting meta-analysis [20].

### Study selection

To be included, studies had to 1) be a case-control or cohort study design with CRC incidence as outcome; 2) provide odds ratio (OR), relative risk (RR) or hazard ratio (HR) estimates with 95% confidence intervals (CI), standard errors (SE) (or information to compute them) of CRC associated with menarcheal age. When multiple publications from the same study were available, we used the publication with the largest number of cases and most applicable information.

**Figure 1:**
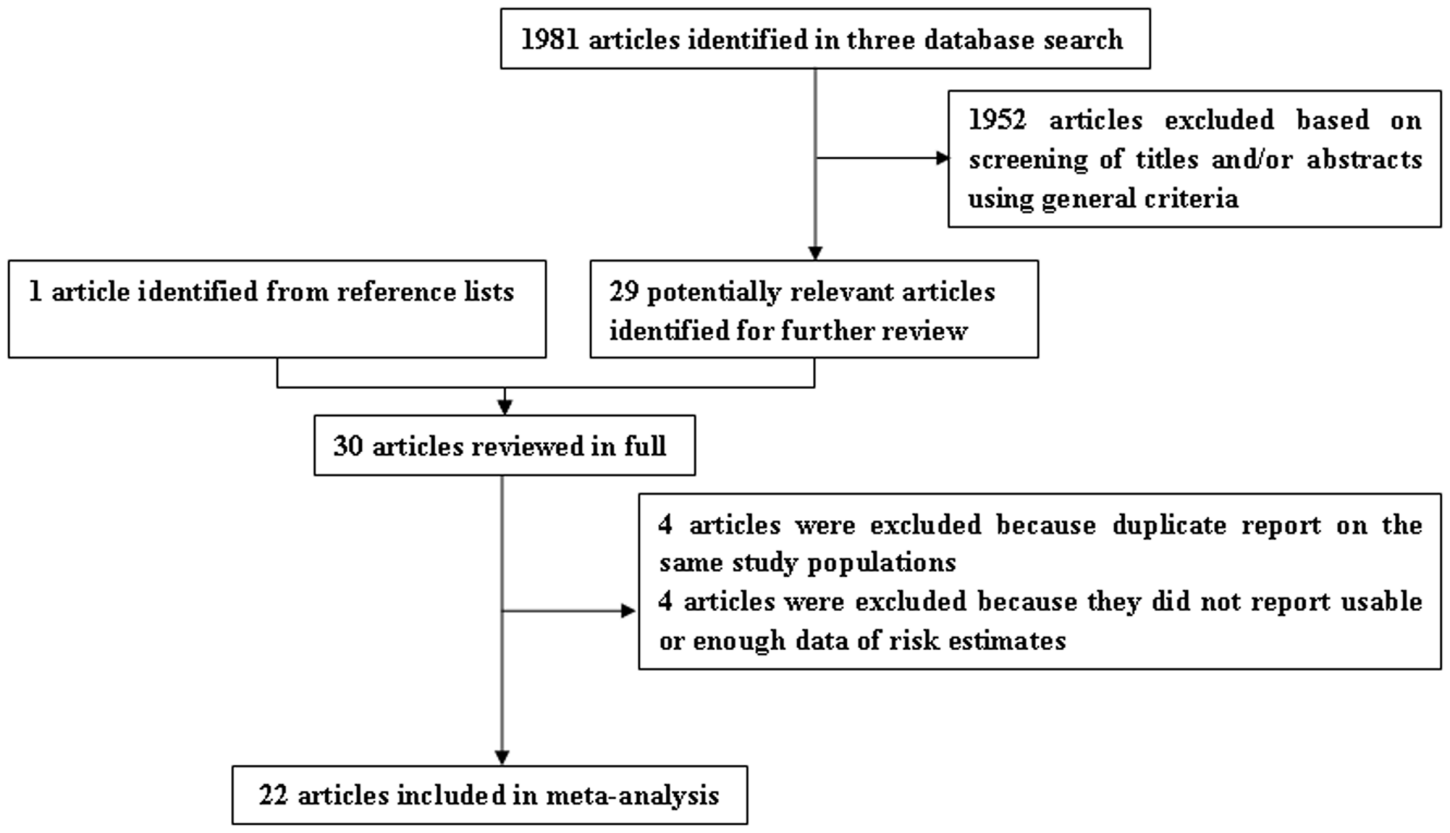
Selection of studies for inclusion in meta-analysis.

We identified 13 prospective cohort studies [9], [14], [15], [16], [18], [19], [21], [22], [23], [24], [25], [26], [27] and 17 case-control studies [10], [11], [12], [13], [17], [28], [29], [30], [31], [32], [33], [34], [35], [36], [37], [38], [39] with data that were potentially eligible for inclusion in the meta-analysis. On this review, one cohort [26] and three case-control studies [34], [35], [36] was duplicate reports from the same study population but we only included two case-control studies [34], [35] in the subgroup analyses because they provided the information of the anatomic cancer site of CRC and cancer subsite of colon, one cohort [27] and three case-control studies [37], [38], [39] were excluded because they did not report usable or enough data of risk estimates. The remaining 22 studies were included in the meta-analysis (Figure 1).

### Data abstraction and quality assessment

For each eligible study, two investigators (C-YL and Q-JW) independently performed the eligibility evaluation, data abstraction, and quality assessment; discrepancies were settled by consensus. Data abstracted from each study included are as follows: the first author's last name, year of publication, study design, the country in which the study was performed, study sample size (numbers of case patients and control subjects or cohort size), duration years of follow-up for cohort studies, exposure assessment and menarcheal age categories, study-specific adjusted ORs or RRs with their 95% CI for the oldest versus youngest category of menarcheal age (if multiple estimates were available, we abstracted the estimate that adjusted for the most covariates), and factors controlled for by matching or in the multivariable model.

To assess study quality, a 9-star system on the basis of the Newcastle-Ottawa Scale [7], [40], [41] was used. A full score was 9 and a quality study was defined as one with a quality score greater than or equal to 7.

### Statistical analysis

The study-specific adjusted RRs were used as the measure of association across studies. Because the absolute risk of CRC is low, we assumed that estimates of ORs from case-control studies and risk, rate or hazard ratios from cohort studies were all valid estimates of the RR and we therefore report all results as the RR for simplicity. For studies that reported results separately for proximal and distal colon or colon and rectal cancer, but not combined, we pooled the results using a fixed-effects model to obtain an overall combined estimate before combining with the rest of the studies [40], [42]. For studies that did not use the category with the youngest menarcheal age as the reference, we used the effective count method proposed by Hamling et al [43] to recalculate the RRs using the stratum with the youngest menarcheal age as the reference.

We evaluated heterogeneity of RRs across studies by using the Cochrane *Q* statistic, where we considered *P*<0.1 to be indicative of statistically significant heterogeneity, and the *I* ^2^ statistic. The summary estimate based on the random effects model [44] or fixed effects model [45] was reported when substantial heterogeneity was detected or not. We used these two effects models to calculate summary RRs and 95% CI for the oldest versus the youngest categories of menarcheal age for the analysis. Heterogeneity between subgroups was evaluated by meta-regression. Subgroup analyses were carried out based on study quality, study design (cohort vs. case-control studies), type of controls within the case-control study (population-based vs. hospital-based controls), geographic location (Europe, America, and Asia), anatomic site of CRC (colon versus rectum cancer), and cancer subsite of colon (proximal versus distal colon cancer). Moreover, we stratified the meta-analysis by potentially important confounders and risk factors. Finally, we performed a sensitivity analysis in which one study at a time was removed and the rest analyzed to evaluate whether the results could have been affected markedly by a single study.

Publication bias was evaluated via Egger's linear regression [46], Begg's rank correlation methods [47] and funnel plots. A *P*-value less than 0.05 for Egger's or Begg's tests was considered representative of significant statistical publication bias. Statistical analyses were performed with Stata (version 11.2; StataCorp, College Station, TX). *P*-values were two sided with a significance level of 0.05.

## Results

### Study characteristics and quality assessment

Characteristics of the 22 included articles are shown in Table S1. Of the 11 prospective cohort studies, six were carried out in the United States [9], [16], [21], [22], [24], [25], two in Japan [18], [23], one each in Korea [14], Europe [15], and Canada [19]. Cohort sizes ranged from 7,381 [25] to 443,909 [14], and the number of CRC cases varied from 68 [25] to 2,153 [14]. The youngest category ranges of menarcheal age varied from 10 [21] to 15 [14] years old, and the highest varied from 14 [9], [19], [22], [24], [25] to 17 [14] years old.

Of the 11 case-control studies, four were carried out in the United States [28], [29], [30], [33], two in Italy [10], [17], one each in Egypt [13], Netherland [12], Sweden [31], China/United States [32], and Greece [11]. The number of CRC cases enrolled in these studies ranged from 86 [11] to 1,488 [29], and the number of control subjects varied from 123 [12] to 4,297 [29]. Control subjects were drawn from the general population in 7 studies [12], [28], [29], [30], [31], [32], [33], hospitals in 4 studies [10], [11], [13], [17]. The youngest category ranges of menarcheal age varied from 11 [10] to 13 [11], [12], [31] years old, and the highest varied from 13 [13], [33] to 19 [32] years old.

Study-specific quality scores are summarized in Tables S2 and S3. The quality scores ranged from 4 to 9 with a median score of 6.5. The median scores of cohort and case-control studies were 8 and 6, separately. High-quality studies (i.e. those studies that had 7 awarded stars) included 8 cohort [9], [14], [15], [18], [19], [21], [22], [23] and 3 case-control studies [12], [29], [30].

### Oldest versus youngest menarcheal age category

**Figure 2:**
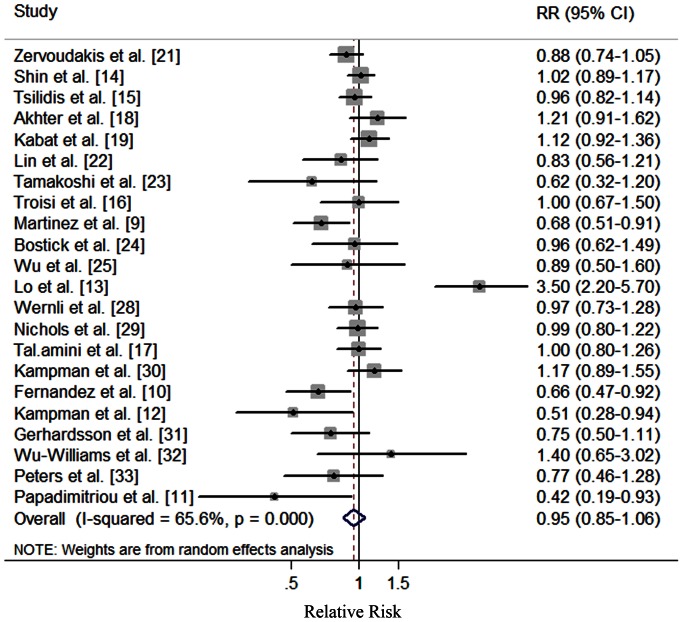
Forest plot (random effects model) of menarcheal age and colorectal cancer risk in overall studies. Squares indicate study-specific relative risks (size of the square reflects the study-specific statistical weight); horizontal lines indicate 95% CIs; diamond indicates the summary relative risk estimate with its 95% CI. CI: confidence interval; RR: relative risk.

The multivariable-adjusted RRs for each study and all studies combined for the oldest versus youngest categories of menarcheal age are shown in Figure 2. In a random-effect meta-analysis of overall studies, we found no association between menarcheal age and CRC risk (RR, 0.95; 95% CI: 0.85–1.06), with significant heterogeneity (*Q* = 61.03, *P*<0.001, *I* ^2^ = 65.6%). Moreover, the similar results were also observed in cohort studies (RR, 0.97; 95% CI: 0.90–1.04) based on a fixed-effect model and case-control studies (RR, 0.95; 95% CI: 0.75–1.21) based on a random-effect model, respectively (Table 1). There was no indication of publication bias with Egger's test (*P* for bias = 0.456) or with Begg's test (*P* for bias = 0.167) and no asymmetry was seen in the funnel plots when inspected visually.

**Table 1:** Summary risk estimates of the association between menarcheal age and colorectal cancer risk.

	No. of	Summary RR	*Q*	*I* ^2^	*P*_h_ *	*P*_h_ **
	studies	(95% CI)	Statistic	Value (%)		
**Overall**	22	0.95 (0.85–1.06)	61.03	65.6	<0.001	—
**Subgroup analyses**						0.742
High quality studies (scores≥7)	11	0.95 (0.86–1.06)	20.35	50.9	0.026	
Study Design						0.848
Cohort studies	11	0.97 (0.90–1.04)	14.33	30.2	0.159	
Case-control studies	11	0.95 (0.75–1.21)	46.66	78.6	<0.001	
Exposure Assessment						0.882
Trained interviewer	7	1.06 (0.75–1.49)	37.98	84.2	<0.001	
Self-administered questionnaire	11	0.96 (0.89–1.03)	15.78	36.6	0.106	
Type of Control Subjects						0.692
Population based	7	0.96 (0.85–1.09)	9.34	35.8	0.155	
Hospital based	4	1.02 (0.51–2.04)	37.05	91.9	<0.001	
Study Population						0.835
Asians	3	1.03 (0.92–1.17)	3.48	42.5	0.175	
Americans	11	0.95 (0.88–1.03)	12.15	17.7	0.275	
Europeans	7	0.90 (0.64–1.27)	43.01	86.0	<0.001	
Anatomic cancer site						0.921
Colon	16	0.96 (0.86–1.08)	23.42	36.0	0.076	
Rectum	11	0.98 (0.86–1.11)	7.03	0	0.722	
Cancer subsite of colon						0.899
Proximal	6	1.11 (0.92–1.34)	6.22	19.6	0.285	
Distal	6	1.09 (0.88–1.35)	2.86	0	0.722	
**Adjustment for important confounders or risk factors**						
Body mass index						0.861
Yes	11	0.98 (0.92–1.05)	15.95	37.3	0.101	
No	11	0.92 (0.69–1.23)	44.70	77.6	<0.001	
Physical activity						0.893
Yes	8	0.94 (0.86–1.03)	12.02	41.8	0.100	
No	14	0.95 (0.80–1.14)	48.17	73.0	<0.001	
Cigarette smoking						0.249
Yes	11	1.02 (0.87–1.18)	41.69	76.0	<0.001	
No	11	0.86 (0.73–1.02)	17.24	42.0	0.069	
Alcohol drinking						0.575
Yes	10	0.95 (0.88–1.02)	12.49	27.9	0.187	
No	12	0.98 (0.78–1.24)	47.14	76.7	<0.001	
Family history of CRC and adenomatous polyposis						0.376
Yes	10	0.90 (0.79–1.03)	17.14	47.5	0.047	
No	12	1.01 (0.85–1.20)	41.57	73.5	<0.001	
OC use						0.814
Yes	17	0.96 (0.83–1.11)	52.48	69.5	<0.001	
No	5	0.93 (0.78–1.01)	8.47	52.8	0.076	

RR: relative risk; CI: confidence interval; CRC: colorectal cancer; OC: oral contraceptive.

* *P* value for heterogeneity within each subgroup.

** *P* value for heterogeneity between subgroups with meta-regression analysis.

### Subgroup and sensitivity analyses

We examined possible differences between risk estimates by various study characteristics. We did not find evidence of heterogeneity and significant association between menarcheal age and CRC risk in pooled estimates by quality of study methodology, exposure assessment, study population, anatomic cancer site, and cancer subsite of colon in which the study was carried out (Table 1). When considering adjustment for potential important confounders or risk factors, we found no significant difference between estimates adjusted and those not adjusted for body mass index (BMI), physical activity, cigarette smoking, alcohol drinking, and other factors.

A sensitivity analysis omitting one study at a time and calculating the pooled RRs for the remainder of the studies showed that the study by Lo et al [13] substantially influenced the pooled RR. After excluding this single study, there was little heterogeneity (*Q* = 32.67, *P* = 0.037, *I* ^2^ = 38.8%), and the RR for the oldest versus youngest category of menarcheal age was 0.93 (95% CI: 0.85–1.01). When we removed three studies in which RRs and 95% CI were not reported but calculated from raw data, the results (RR, 0.98; 95% CI: 0.87–1.09) were similar.

## Discussion

This, to our knowledge, is the first meta-analysis to explore the association between menarcheal age and CRC risk. In the present study, we found that menarcheal age is not associated with CRC risk. Additionally, there was no association between menarcheal age and CRC risk in the subgroup meta-analyses.

The exact biologic mechanisms underlying the association between menarcheal age and decreased risk of CRC are not fully understood, but certainly involve alterations in the metabolism of endogenous hormones, including estrogen, estradiol, IGFs. Age at menarche is an indicator of not only the duration of exposure to cyclic ovarian function but also the sex hormone change among the period of childhood and adolescence. Early menarche is associated with a more rapid onset of ovulatory cycles and a tendency to sustain higher levels of luteal phase estradiol [48]. Experimental studies provided evidence that in human CRC cell lines, estradiol has been shown to activate the mitogen-activated protein kinase cascade, a pathway that plays a key role in the stimulation of DNA and protein synthesis, which induces cell growth and proliferation [49], [50]. Moreover, CRC tissue was found to have higher levels of estradiol activity compared with nonmalignant colorectal tissue [51], [52], and a cross-sectional study of colon cancer patients demonstrated that colon carcinoma tissue had a statistically significant twofold higher level of total estrogen compared with normal colon mucosa [53]. On the other hand, estrogen, may play a protective role in colorectal carcinogenesis either indirectly by decreasing in circulating bile acid concentration levels and down regulation of IGF-I or directly by regulating cell growth in the colonic epithelium and inhibiting cell proliferation of colorectal tumors by binding to the estrogen receptor [4]. Issa et al [54] demonstrated that the estrogen receptor may act as a tumor suppressor which methylation of the estrogen receptor increases with age in individuals without colonic tumors, but estrogen receptor methylation is almost universally present in individuals with colonic tumors. Even though the results of previously mentioned experimental studies are suggestive of a reduction in CRC risk, the meta-analysis of epidemiological studies still have an insufficient evidence to draw definite conclusions about this issue.

Although we yielded the similar association in the subgroup analyses of study design, type of control subjects in case-control studies and exposure assessment, the heterogeneity were rather different among these subgroup analyses (Table 1), which could be explained by the quality of the study methodologies included in the current studies. As a meta-analysis of epidemiological studies, it is prone to bias (e.g., recall and selection bias) inherent in the original studies. Cohort studies are less susceptible to bias than case-control studies because, in the prospective design, information on exposures is collected before the diagnosis of the disease. Compared with case-control studies, cohort studies provided more detailed information of adjustment for confounders. Inadequate control for confounders may bias the results in either direction, toward exaggeration or underestimation of risk estimates. Furthermore, after the assessment the quality of these two kinds of study, case-control studies had a lower median score than cohort studies, namely, 6 and 8, respectively.

Considering that the predominant premenopausal profile of endogenous female sex hormones derived from the ovaries modifies the risk of female CRC through increased excretion of bile acids and the effect of hyperinsulinemia on the risk of CRC may predominate in postmenopausal women with low levels of female sex hormone [23], [55]. Several studies suggested that the relationships between menarcheal age and CRC might be modified by the menopause status. However, only 1 [18] and 4 [14], [18], [21], [23] included studies reported the association between menarcheal age and CRC risk in premenopausal and postmenopausal status, respectively. Given this, future studies are warranted to focus on this issue. On the other hand, some genetic loci were revealed to be associated with menarcheal age by several genome wide assoscation study (GWAS) [56], [57], [58], [59]. Moreover, the interaction between environments and genetic factors has also been considered by several research [60], [61]. Therefore, future epidemiological studies should focus on whether genetic factors might modify the menarcheal age in the development of CRC.

Our meta-analysis has several strengths. This study is the large sample size with more than 15,479 cases and 239,957 subjects which should have provided sufficient statistical power to detect the putative association between menarcheal age and CRC. Moreover, our study is the thorough statistical analyses considering a number of subgroups. Sensitivity analyses were also carried out to investigate whether any particular study explained the results and the findings still consistent with the overall results. However, several limitations to this study also should be addressed. First, information on menarcheal age and other reproductive variables were based on a self-administered baseline questionnaire or trained interviewer, and none of the study demonstrated a repeated measurement that was initially answered by the participants. Thus some non-differential misclassification of participants was inevitable and would probably lead to an underestimation of the results. Secondly, a meta-analysis is not able to solve problems with confounding factors that could be inherent in the included studies, which may introduce bias in an unpredictable direction. Although most studies adjusted for some known risk factors for CRC, residual or unknown confounding cannot be excluded as a potential explanation for the observed findings. Later menarcheal age tends to be associated with lower levels of BMI [62], higher prevalence of smoking [63], later ages at first alcohol drinking [64], and higher physical activity [65]. However, although the results of meta-regression analyses indicated that the adjustment for these confounders was not a source of heterogeneity, only two studies [9], [22] adjusted for all major potential confounding and risk factors. Last, significant heterogeneity and possible publication bias must be considered. There was significant heterogeneity for all studies combined (*Q* = 61.03, *P*<0.001, *I* ^2^ = 65.6%) in the pooled analysis of menarcheal age; however, this could be at least partially explained by differences in study quality, study design, exposure assessment, study population and adjustment for potential confounders and risk factors (Table 1). Publication bias can be a problem in meta-analyses of published studies; however, we found no statistical evidence of publication bias in this meta-analysis, and there was also no asymmetry in the funnel plots when inspected visually.

In summary, the results of this meta-analysis provide no evidence that later menarcheal age is associated with CRC risk. Given the limited number of studies reported the association among pre-and post-menopause status, future cohort and well-designed case-control studies should extend this issue.

## Supporting Information

Table S1 **Characteristics of studies of menarcheal age and colorectal cancer risk.**
(DOC)

Table S2 **Methodological quality of the prospective studies included in the meta-analysis.**
(DOC)

Table S3 **Methodological quality of the case-control studies included in the meta-analysis.**
(DOC)
