# Supplementary material for: Age at Menarche and Risk of Colorectal Cancer: A Meta-Analysis
Source: PLoS One. 2013 Jun 6;8(6):e65645. doi: 10.1371/journal.pone.0065645 (PMC3675201; doi:10.1371/journal.pone.0065645)
Supplement: Table S1 — Characteristics of studies of menarcheal age and colorectal cancer risk. (DOC) [file pone.0065645.s001.doc]

**Table S1 Characteristics of studies of menarcheal age and colorectal cancer risk**

| **First author, publication year** **(reference), Country,** **Study design** | **Cases/subject (age), duration of follow up** | **Menarcheal age categories (exposure/case assessment)** | **RR/OR (95%CI)** | **Adjusted potential factors** | | | | | |
| --- | --- | --- | --- | --- | --- | --- | --- | --- | --- |
|  |  |  |  | **BMI** | **OC use** | **PA** | **Cigarette smoking** | **Alcohol drinking** | **Family history of CRC or adenomatous polyposis** |
| **Prospective study** |  |  |  |  |  |  |  |  |  |
| Zervoudakis [21], 2011, USA, CS | 2,014/214,162 (50-71y), 8.2y | CRC ≥15 vs. <10  (Self-questionnaire/cancer registry) | 0.88 (0.74-1.05) | ✓ | — | ✓ | ✓ | ✓ | ✓ |
| Shin † [14], 2011, Korea, CS | 2,153/443,909 (30-80y), 12y | CRC ≥17 vs. <15  CC ≥17 vs. <15  RC ≥17 vs. <15  (Self-questionnaire/cancer registry) | 1.02 (0.89-1.17)  0.96 (0.80-1.16)  1.10 (0.89-1.35) | ✓ | — | — | ✓ | ✓ | — |
| Tsilidis [15], 2010, European, CS | 1,878/337,802 (35-70y), 9y | CRC ≥15 vs. <12  (Self-questionnaire/medical records) | 0.96 (0.82-1.14) | ✓ | — | ✓ | ✓ | ✓ | — |
| Akhter [18], 2008, Japan, CS | 538/48,511 (40-69y), 12y | CRC ≥16 vs. ≤13  CC ≥16 vs. ≤13  RC ≥16 vs. ≤13  (Self-questionnaire/cancer registry) | 1.21 (0.91-1.62)  1.25 (0.88-1.79)  1.14 (0.69-1.87) | ✓ | — | ✓ | ✓ | ✓ | ✓ |
| Kabat [19], 2008, Canada, CS | 1,142/89,835 (40-59y), 16.4y | CRC ≥14 vs. <12  CC ≥14 vs. <12  RC ≥14 vs. <12  (Self-questionnaire/cancer registry) | 1.12 (0.92-1.36)  1.17 (0.93-1.48)  0.97 (0.69-1.35) | ✓ | ✓ | — | ✓ | — | — |
| Lin [22], 2007, USA, CS | 267/39,680 (≥45y), 11y | CRC ≥14 vs. <12  CC ≥14 vs. <12  RC ≥14 vs. <12  (Self-questionnaire/medical records) | 0.83 (0.56-1.21)  0.83 (0.53-1.31)  0.93 (0.44-1.97) | ✓ | ✓ | ✓ | ✓ | ✓ | ✓ |
| Tamakoshi [23], 2004, Japan, CS | 207/38,420 (40-79y), 7.6y | CC ≥16 vs. ≤12  (Self-questionnaire/cancer registry) | 0.62 (0.32-1.20) | ✓ | — | ✓ | ✓ | ✓ | ✓ |
|  |  |  |  | ***(Continued)*** | | | | | |

**Table S1**

**(Continued)**

| **First author, publication year (reference), Country, Study design** | **Cases/subject (age), duration of follow up** | **Menarcheal age categories (exposure/ case assessment)** | **RR/OR (95%CI)** | **Adjusted potential factors** | | | | | |
| --- | --- | --- | --- | --- | --- | --- | --- | --- | --- |
|  |  |  |  | **BMI** | **OC use** | **PA** | **Cigarette smoking** | **Alcohol drinking** | **Family history of CRC or adenomatous polyposis** |
| Troisi [16], 1997, USA, CS | 203/57,529 (31-90y), 10y | CRC ≥15 vs. ≤11  CC ≥15 vs. ≤11  RC ≥15 vs. ≤11  (Interviewer/medical record) | 1.00 (0.67-1.50)  1.10 (0.60-2.10)  0.62 (0.25-1.60) | — | — | — | — | — | — |
| Martinez † [9], 1997, USA, CS | 501/89,448 (30-55y), 12y | CRC ≥14 vs. <12  CC ≥14 vs. <12  RC ≥14 vs. <12  (Self-questionnaire/cancer registry) | 0.68 (0.51-0.91)  0.63 (0.46-0.88)  0.91 (0.47-1.77) | ✓ | ✓ | ✓ | ✓ | ✓ | ✓ |
| Bostick [24], 1994, USA, CS | 212/35,215 (55-69y), 5y | CC >14 vs. <13  (Self-questionnaire/cancer registry) | 0.96 (0.62-1.49) | — | — | — | — | — | — |
| Wu [25], 1987, USA, CS | 68/7,381 (N/A), 4.5y | CRC ≥14 vs. ≤12  (Self-questionnaire/medical record) | 0.89 (0.50-1.60) | — | — | — | — | — | — |
| **Case-control study** |  |  |  |  |  |  |  |  |  |
| Lo [13], 2010, Egypt, HC-CS | 200/203 (N/A) | CRC ≥13 vs. ≤12  (Trained interviewer/medical record) | 3.5 (2.2-5.7) | — | — | — | — | — | — |
| Wernli [28], 2009, USA, PC-CS | 1,014/1,064 (50-74y) | CRC ≥14 vs. <12  CC ≥14 vs. <12  RC ≥14 vs. <12  (Interviewer/cancer registry) | 0.97 (0.73-1.28)  0.99 (0.73-1.35)  0.62 (0.37-1.04) | ✓ | ✓ | — | ✓ | ✓ | ✓ |
|  |  |  |  | ***(Continued)*** | | | | | |

**Table S1**

**(Continued)**

| **First author, publication year (reference), Country, Study design** | **Cases/subject (age), duration of follow up** | **Menarcheal age categories (exposure/ case assessment)** | **RR/OR (95%CI)** | **Adjusted potential factors** | | | | | |
| --- | --- | --- | --- | --- | --- | --- | --- | --- | --- |
|  |  |  |  | **BMI** | **OC use** | **PA** | **Cigarette smoking** | **Alcohol drinking** | **Family history of CRC or adenomatous polyposis** |
| Nichols [29], 2005, USA, PC-CS | 1,488/4,297 (20-74y) | CRC >14 vs. <12  CC >14 vs. <12  RC >14 vs. <12  (Trained interviewer/cancer registry) | 0.99 (0.80-1.22)  1.11 (0.87-1.43)  0.95 (0.67-1.34) | ✓ | — | — | ✓ | — | ✓ |
| Talamini [17], 1998, Italy, HC-CS | 828/2,081 (median, 62/56y) | CC ≥14 vs. <12  RC ≥14 vs. <12  (Trained interviewer/medical record) | 1.06 (0.79-1.40)  0.92 (0.64-1.32) | — | — | ✓ | — | — | — |
| Kampman [30], 1997, USA, PC-CS | 894/1,120 (mean, 64.6/64.8y) | CC ≥14 vs. <12  (Interviewer/medical records) | 1.17 (0.89-1.55) | ✓ | — | ✓ | — | — | ✓ |
| Fernandez § [10], 1996, Italy, HC-CS | 709/992 (median, 61/58y) | CRC ≥15 vs. ≤11  (Trained interviewer/medical record) | 0.66 (0.47-0.92) | — | — | — | — | — | — |
| Kampman [12], 1994, Netherland, PC-CS | 102/123 (mean, 61.3/61.2y) | CC ≥14 vs. ≤13  (Trained interviewer/cancer registry) | 0.51 (0.28-0.94) | — | — | — | — | — | ✓ |
| Gerhardsson [31], 1992, Sweden, PC-CS | 299/276 (41-80y) | CC ≥13 vs. <13  RC ≥13 vs. <13  (Self-questionnaire/cancer registry) | 0.7 (0.4-1.2)  0.8 (0.4-1.3) | — | — | — | — | — | — |
| Wu-Williams § [32], 1991, China/USA, PC-CS | 395/1,112 (≥20y) | CC ≥19 vs. <12  RC ≥19 vs. <12  (Trained interviewer/cancer registry) | 1.20 (0.38-3.77)  1.55 (0.59-4.03) | — | — | — | — | — | — |
| Peter [33], 1990, USA, PC-CS | 327/327 (N/A) | CC >13 vs. <12  (Interviewer/cancer registry) | 0.77 (0.46-1.28) | — | — | ✓ | — | ✓ | ✓ |
|  |  |  |  | ***(Continued)*** | | | | | |

**Table S1**

**(Continued)**

| **First author, publication year (reference), Country, Study design** | **Cases/subject (age), duration of follow up** | **Menarcheal age categories (exposure/ case assessment)** | **RR/OR (95%CI)** | **Adjusted potential factors** | | | | | |
| --- | --- | --- | --- | --- | --- | --- | --- | --- | --- |
|  |  |  |  | **BMI** | **OC use** | **PA** | **Cigarette smoking** | **Alcohol drinking** | **Family history of CRC or adenomatous polyposis** |
| Papadimitriou § [11], 1984, Greece, HC-CS | 86/369 (median, 47/44y) | CRC ≥14 vs. ≤13  (Interviewer/medical records) | 0.42 (0.19-0.93) | — | — | — | — | — | — |

RR: relative risk; OR: odds ratio; CI: confidence interval; HC-CS: hospital-based case-control study; PC-CS: population-based case-control study; CS: cohort study; N/A: not available; BMI: body mass index; OC: oral contraceptive; PA: physical activity; CRC: colorectal cancer; CC: colon cancer; RC: rectal cancer.

† Recalculate the RR by the method proposed by Hamling et al.

§ Odds ratio and 95% CI calculated from published data using EpiCalc 2000.
