# Supplementary material for: Age at Menarche and Risk of Colorectal Cancer: A Meta-Analysis
Source: PLoS One. 2013 Jun 6;8(6):e65645. doi: 10.1371/journal.pone.0065645 (PMC3675201; doi:10.1371/journal.pone.0065645)
Supplement: Table S2 — Methodological quality of the prospective studies included in the meta-analysis. (DOC) [file pone.0065645.s002.doc]

**Table S2. Methodological quality of cohort studies included in the meta-analysis***

| **First author,**  **publication year**  **[reference]** | **Representativeness**  **of the exposed cohort** | **Selection of the unexposed**  **cohort** | **Ascertainment**  **of exposure** | **Outcome of interest**  **not present**  **at start of study** | **Control for**  **important factor or additional factor^†^** | **Assessment of outcome** | **Follow-up**  **long enough for outcomes**  **to occur^‡^** | **Adequacy of**  **follow-up**  **of cohorts^§^** | **Total quality**  **scores** |
| --- | --- | --- | --- | --- | --- | --- | --- | --- | --- |
| Zervoudakis [21], 2011 | ⚝ | ⚝ | ⚝ | ⚝ | ⚝⚝ | ⚝ | — | ⚝ | 8 |
| Shin [14], 2011 | ⚝ | ⚝ | ⚝ | ⚝ | ⚝ | ⚝ | ⚝ | ⚝ | 8 |
| Tsilidis [15], 2010 | ⚝ | ⚝ | ⚝ | ⚝ | ⚝ | ⚝ | — | ⚝ | 7 |
| Akhter [18], 2008 | ⚝ | ⚝ | ⚝ | ⚝ | ⚝⚝ | ⚝ | ⚝ | ⚝ | 9 |
| Kabat [19], 2008 | ⚝ | ⚝ | ⚝ | — | ⚝⚝ | ⚝ | ⚝ | ⚝ | 8 |
| Lin [22], 2007 | ⚝ | ⚝ | ⚝ | ⚝ | ⚝⚝ | ⚝ | ⚝ | ⚝ | 9 |
| Tamakoshi [23], 2004 | ⚝ | ⚝ | ⚝ | ⚝ | ⚝⚝ | ⚝ | — | ⚝ | 8 |
| Troisi [16], 1997 | ⚝ | ⚝ | ⚝ | ⚝ | — | — | — | ⚝ | 5 |
| Martinez [9], 1997 | ⚝ | ⚝ | ⚝ | ⚝ | ⚝⚝ | ⚝ | ⚝ | ⚝ | 9 |
| Bostick [24], 1994 | ⚝ | ⚝ | ⚝ | ⚝ | — | ⚝ | — | ⚝ | 6 |
| Wu [25], 1987 | ⚝ | ⚝ | ⚝ | ⚝ | — | ⚝ | — | ⚝ | 6 |

* A study could be awarded a maximum of one star for each item except for the item Control for important factor or additional factor. The definition/explanation of each column of the Newcastle-Ottawa Scale is available from (http://www.ohri.ca/programs/clinical_epidemiology/oxford.htm.).

† A maximum of 2 stars could be awarded for this item. Studies that controlled for body mass index (BMI) received one star, whereas studies that controlled for other important confounders such as use of oral contraceptive (OC), family history of colorectal cancer received an additional star.

‡ A cohort study with a follow-up time >10 y was assigned one star.

§ A cohort study with a follow-up rate >75% was assigned one star.
