# Supplementary material for: Age at Menarche and Risk of Colorectal Cancer: A Meta-Analysis
Source: PLoS One. 2013 Jun 6;8(6):e65645. doi: 10.1371/journal.pone.0065645 (PMC3675201; doi:10.1371/journal.pone.0065645)
Supplement: Table S3 — Methodological quality of the case-control studies included in the meta-analysis. (DOC) [file pone.0065645.s003.doc]

**Table S3. Methodological quality of case-control studies included in the meta-analysis***

| **First author,**  **publication year [reference]** | **Adequate definition of cases** | **Representativeness of cases** | **Selection of control subjects** | **Definition of control subjects** | **Control for important factor or additional factor^†^** | **Exposure assessment** | **Same method of ascertainment for all subjects** | **Non-response Rate^‡^** | **Total quality scores** |
| --- | --- | --- | --- | --- | --- | --- | --- | --- | --- |
| Lo [13], 2010 | ⚝ | ⚝ | — | ⚝ | — | ⚝ | ⚝ | ⚝ | 6 |
| Wernli [28], 2009 | ⚝ | ⚝ | — | ⚝ | ⚝⚝ | — | ⚝ | — | 6 |
| Nichols [29], 2005 | ⚝ | ⚝ | ⚝ | ⚝ | ⚝⚝ | ⚝ | ⚝ | — | 8 |
| Talamini [17], 1998 | ⚝ | ⚝ | — | ⚝ | — | ⚝ | ⚝ | ⚝ | 6 |
| Kampman [30], 1997 | ⚝ | ⚝ | ⚝ | ⚝ | ⚝⚝ | — | ⚝ | — | 7 |
| Fernandez [10], 1996 | ⚝ | ⚝ | — | — | — | ⚝ | ⚝ | ⚝ | 5 |
| Kampman [12], 1994 | ⚝ | ⚝ | ⚝ | ⚝ | ⚝ | ⚝ | ⚝ | — | 7 |
| Gerhardsson [31], 1992 | ⚝ | ⚝ | ⚝ | — | — | ⚝ | ⚝ | ⚝ | 6 |
| Wu-Williams [32], 1991 | ⚝ | ⚝ | ⚝ | — | — | ⚝ | ⚝ | — | 5 |
| Peter [33], 1990 | ⚝ | ⚝ | ⚝ | ⚝ | — | — | ⚝ | — | 5 |
| Papadimitriou [11], 1984 | ⚝ | ⚝ | — | — | — | — | ⚝ | ⚝ | 4 |

* A study could be awarded a maximum of one star for each item except for the item Control for important factor or additional factor. The definition/explanation of each column of the Newcastle-Ottawa Scale is available from (http://www.ohri.ca/programs/clinical_epidemiology/oxford.htm.).

† A maximum of 2 stars could be awarded for this item. Studies that controlled for body mass index (BMI) received one star, whereas studies that controlled for other important confounders such as use of oral contraceptive (OC), family history of colorectal cancer received an additional star.

‡ One star was assigned if there was no significant difference in the response rate between control subjects and cases by using the chi-square test (*P*>0.05).
